# Planning abilities of children aged 4 years and 9 months to 8 ½ years: Effects of age, fluid intelligence and school type on performance in the Tower of London test

**DOI:** 10.1590/S1980-57642009DN20100006

**Published:** 2008

**Authors:** Leandro Fernandes Malloy-Diniz, Cláudia Cardoso-Martins, Elaine Pacheco Nassif, Angela Maria Levy, Wellington Borges Leite, Daniel Fuentes

**Affiliations:** 1 Universidade FUMEC – Grupo de pesquisa em Neuropsicologia e Neurociências. Nova Lima. Belo Horizonte. Brasil.; 2 Departamento de Psicologia da Faculdade de Filosofia e Ciências Humanas da Universidade Federal de Minas Gerais.; 3 GEDAHI – (Grupo de Estudos dos Distúrbios da Aprendizagem, Atenção e Hiperatividade).; 4 Unidade de Psicologia e Neuropsicologia do Hospital das Clínicas da USP, São Paulo, Brasil.

**Keywords:** planning, cognition, neuropsychology, private vs. public school, socioeconomic status, child development, planejamento, cognição, neuropsicologia, escola particular vs. escola pública, fatores socioeconômicos, desenvolvimento infantil

## Abstract

The present study investigated the relationship between age and one type of environmental factor, namely, type of school (i.e., private vs. public), and the development of mental planning ability, as measured by the Tower of London (TOL) test.

**Methods:**

Participants comprised 197 public and 174 private school students, ranging in age from 4 years and 9 months to 8 years and 6 months. Besides the TOL test, students were administered Raven's Colored Matrices.

**Results:**

Results confirmed the findings of previous studies that both age and school type are important predictors of mental planning. Furthermore, results also suggest that the relationship between type of school and mental planning ability cannot be accounted for by differences in students' fluid intelligence.

**Conclusion:**

In the present study, the TOL test continued to differentiate public from private school students, even after we controlled for the effect of differences on the Raven test.

## Abstract (pt)

O presente estudo investigou a relação entre idade, e um tipo de fator ambiental, o tipo de ambiente escolar (público x privado) e o desenvolvimento das habilidades mentais de planejamento medidas pelo teste da Torre de Londres (TOL).

**Métodos:**

Os participantes foram 197 estudantes de escolas públicas e 174 de escolas privadas com idade variando de 4 anos e 9 meses a 8 anos e 6 meses. Além do TOL, foi administrado o teste das Matrizes Coloridas de Raven.

**Resultados:**

Os resultados confirmam os achados de estudos prévios de que tanto a idade e o tipo de escola são importantes preditores das habilidades de planejamento. Além disso, eles também sugerem que a relação entre o tipo de escola e as habilidades de planejamento não podem ser explicadas por diferenças na inteligência fluída dos estudantes.

**Conclusão:**

No presente estudo, o teste da torre de Londres diferenciou crianças de escolas públicas e privadas mesmo após ter sido controlado o efeito das diferenças medidas pelo teste das Matrizes Coloridas de Raven.

Mental planning is defined as a series of activities entailing 1) identification of the steps needed to reach a given goal; 2) analysis of the alternative means of solving the problem or problems at hand; and 3) selection of the approach that seems to be the most advantageous.^1^ Not surprisingly, it is a complex ability that presumes the integrity of various cognitive processes such as inhibitory control, working memory, and set-shifting. These processes are known in the literature as executive functions, and are subserved by circuits encompassing the prefrontal cortex, basal ganglia and the cerebellum.^2^ There is evidence that impairments in these processes are related to developmental disorders such as ADHD, Tourette Syndrome and Autism, and can affect normal performance on tasks that assess planning ability.^3^

Both age and fluid intelligence (i.e., the ability to reason about and solve novel problems)^4^ have been shown to be important predictors of performance on tasks measuring planning ability (e.g., Tower of London and Tower of Hanoi).^4^ As reviewed briefly below, the results of recent studies suggest that environmental variables also exert a powerful influence on the development of executive functions, including mental planning.

Several studies have demonstrated a strong association between socioeconomic status (SES) and cognitive development.^5-7^ Recently, Noble et al.^6^ suggested that the influence of SES may vary as a function of the neurocognitive system involved. Specifically, they suggested that neurocognitive systems that undergo protracted developmental periods may be particularly susceptible to environmental influences. Accordingly, they found that performance on tasks subserved by the frontal cortex and the left perisylvian systems, both of which involve prolonged periods of postnatal development, correlated strongly with SES. In contrast, performance on tasks assessing neurocognitive systems with shorter periods of postnatal development, such as the occipitotemporal-visual and the parietal systems was more immune to SES differences.

SES is a complex variable that encompasses a multitude of influences. Noble, Norman and Farah^5^ have shown that parental education is the most predictive constituent of SES for the development of executive functions and language skills, suggesting that home variables that are likely to be affected by parental education, such as, for example, the home literacy environment and parent-child styles of interaction, are important influences on the development of mental planning and language skills. Recent research by Ardilla et al.^7^ with Mexican and Colombian children suggests that the school environment also has an important impact on the development of mental planning. In particular, these researchers have shown that the type of school attended by the child – private vs. public – accounts for variations in tasks measuring executive functions and mental planning, above and beyond differences in parental education. In the present study, we extend the work by Ardilla et al. by investigating whether the influence of the type of school is independent of variations in children's fluid intelligence.

## Methods

### Participants

Participants comprised 197 children (101 girls, 96 boys) enrolled in private schools, and 174 (86 girls, 88 boys) enrolled in public schools in a major Brazilian city. At both schools, children were divided into four age groups: 4 years, 9 months to 5 years, 5 months, 29 days; 5 years, 6 months to 6 years, 5 months, 29 days; 6 years, 6 months to 7 years, 5 months, 29 days; and, finally, 7 years, 6 months to 8 years, 5 months, 29 days (Table 1). At the time of the assessment, 82 participants were enrolled in preschool classes (39 private and 43 public school students), 227 in 1^st^ grade classes (129 private and 108 public school students), and 52 in 2^nd^ grade classes (29 private and 23 public school students).

**Table 1:** Mean age (in months) and number of girls and boys as functions of age group and type of school.

Age group	Private Schools						Public Schools				
	Girls			Boys			Girls			Boys	
	N	Mean age (± SD)		N	Mean age (± SD)		N	Mean age (± SD)		N	Mean age (± SD)
4 years; 9 months to 5 years; 5 months; and 29 days	22	60.5 (± 2.7)		17	61.4 (± 2.2)		17	60.1 (± 1.8)		26	60.8 (± 2.3)
5 years; 6 months to 6 years; 5 months; and 29 days	16	71.6 (± 3.1)		19	70.8 (± 3.7)		22	71.9 (± 3.0)		28	71.1 (± 3.4)
6 years; 6 months to 7 years; 5 months; and 29 days	29	84.5 (± 3.2)		27	83.5 (± 3.5)		19	84.2 (± 3.3)		22	83.1 (± 3.0)
7 years; 6 months to 8 years; 5 months; and 29 days	34	94.5 (± 2.1)		33	94.3 (± 2.2)		28	93.2 (± 2.1)		12	93.5 (± 1.9)

Children were chosen randomly from schools' registration records. To participate in the study, children had to present no history of neurological and/or psychological impairments. In addition, only children who were enrolled in age appropriate classes were included in the study.

### Instruments

*The Tower of London (TOL)* task^8^ was used to assess mental planning. The Krikorian's^9^ task is performed on a wooden board consisting of three pegs of descending height, on which three balls (one green, one red, and one blue) can be arranged, three on the first peg, two on the second, and one on the third. In each of 12 different problems, the participant is asked to arrange the balls on the test board according to a picture display. The board is always presented with the balls arranged in the same configuration, that is, with the green and blue balls placed on the first peg (the green ball under the blue one), and the blue ball on the second peg. The participant is told that only one ball can be moved at a time, and that he or she should try to solve the problem with the fewest ball movements possible. For each problem, the participant gets three points if the problem is solved in the first trial, two points if the problem is solved in the second trial, one point if it is solved in the third and, finally, zero if he or she cannot solve the problem within the three trials. The score corresponds to the total sum of points.

*Raven's Colored Matrices*^10^ (RCM) were used to assess fluid intelligence. The test consists of three sets of 12 problems each. For each problem, the child is shown an incomplete design or matrix with six figures printed underneath it. The child's task consists of pointing to the figure that completes the matrix. For each correct response, the child earns 1 point. The child's score corresponds to the total number of points (Maximum score=36 points).

The children were tested individually in a quiet location at their school. The TOL test was always administered before the RCM.

Statistics were calculated with the SPSS 15.0 (Statistical Package for Social Sciences). Student's t-tests were used to compare public and private school student performance on the TOL and Raven's measures, while Pearson's correlation was used to calculate the association between performance on the TOL test and performance on RCM. Finally, an analysis of variance (ANOVA) was calculated to test the effects of age, type of school, and gender, as well as any interaction between these factors, with scores on the Raven test as covariates. Tukey HSD multiple comparison post-hoc tests were used as appropriate.

This study was approved by the Research Ethics Committee of FUMEC University (REF263/2007), Belo Horizonte, MG, Brazil.

## Results

Table 2 lists the results for the TOL and the RCM tests, separately for the private and public schools, and for the four age ranges. As can be seen from this table, scores on both tasks varied across the two types of school. Specifically, private school students performed significantly better than public school students on both the TOL (*t*(369)= 5,55, *p*<.001) and the RCM (*t* (369)=10,09, *p*<.001). In addition, performance on RCM correlated significantly with performance on the TOL (*r* =.42, *p*<.001). In view of these results, the scores on the TOL were submitted to an ANOVA with gender, age, and type of school as between-subjects factors, using scores on RCM.

**Table 2:** Mean scores on the TOL and Raven tests as functions of age, gender, and type of school.

Age group	Gender	Private school						Public school				
		Raven Raw score			TOL score			Raven Raw score			TOL score	
		Mean	SD		Mean	SD		Mean	SD		Mean	SD
4 years; 9 months to 5 years; 5 months; and 29 days	Girls (n=39)	13.9	2.6		24.5	5.2		14.2	3.2		26.1	2.3
	Boys (n=43)	17.4	2.5		28.7	2.8		2.8	2.8		2.8	3.3
	Total (n=82)	15.4	3.1		26.4	4.7		14.1	2.4		26.4	3.0
5 years; 6 months to 6 years; 5 months; and 29 days	Girls (n=38)	19.8	4.0		29.4	29.4		29.4	29.4		26.6	4.2
	Boys (n=47)	19.4	3.9		29.4	3.2		15.3	2.0		26.9	5.6
	Total (n=85)	19.5	3.9		29.4	3.2		15.8	2.4		26.8	5.0
6 years; 6 months to 7 years; 5 months; and 29 days	Girls (n=48)	20.9	3.9		28.9	3.8		17.4	3.6		25.5	4.8
	Boys (n=49)	22.4	4.4		28.6	3.5		17.3	2.8		27.1	4.7
	Total (n=97)	21.7	4.2		28.8	3.6		17.3	3.2		26.4	4.8
7 years; 6 months to 8 years; 5 months; and 29 days	Girls (n=62)	25.9	3.4		31.4	3.8		19.5	3.7		28.3	3.0
	Boys (n=45)	25.9	3.8		31.7	2.6		20.4	4.8		28.8	2.2
	Total (n=107)	25.9	3.6		31.5	3.2		19.8	4.0		28.5	2.8

There was a trend for gender to be significant (*F*(1, 354) =3.81, *p*=052). This resulted from a small difference favoring boys (Mean=27.92, SD=4.44, for the girls and M= 28.54, SD=4.06, for the boys). Both age and school type had a significant effect on TOL performance (*F*(3, 354)=4, 31, *p*=.005, for age and *F*(1, 354)=9.18, *p*=.003, for school type. As can be seen from Table 1, performance on the TOL improved with age and was higher for private school students than for public school students. Tukey HSD multiple comparisons showed that the oldest age group performed significantly better than all other age groups (all *ps*<.001). There was also a trend for a significant difference between the first and the second age groups. Finally, no significant interactions were found among age and gender, age and school type, gender and school type, or gender, age and school type.

## Discussion

The main goal of the present study was to replicate Ardilla et al.^7^ finding that private school students perform significantly better than public school students on tasks measuring mental planning. In particular, we were interested in investigating if this effect is independent of differences in private and public school students' fluid intelligence.

In the present study, performance on RCM was significantly correlated with performance on the TOL, confirming Zook et al.^4^ finding that fluid intelligence is an important correlate of mental planning. Furthermore, the public school students performed significantly more poorly than the private school students on both the TOL and the RCM. Notwithstanding these effects, our results showed that the effect of type of school on TOL performance is not mediated by differences in students' fluid intelligence.

These results extend Ardilla et al. findings, and suggest that characteristics of the school environment may contribute to SES differences in neurocognitive development. Several differences between Brazilian private and public schools could account for the results of the present study. For example, there is evidence^11^ of a difference favoring private schools as opposed to public schools in terms of both physical and professional resources. A more in-depth assessment of children's school environments, including teacher-student styles of interaction, is needed to help us disentangle the factors that influence the development of planning ability. Likewise, it will be important to assess differences in students' motivation and beliefs about intelligence,^12^ and how these variations are related to variations in students' planning ability, as well as to school environment variables. Very likely, the results of this research will have important educational implications. Future studies should also explore the effect of possible interactions between type of school attendance and other environmental variables. For example, it is often the case that private school students begin to attend school at an earlier age than public school students, and it is possible that this factor accounts for a substantial portion of the variation observed in TOL performance.

The distribution of the TOL scores was slightly negatively skewed, confirming Portela et al.^13^ suggestion that the TOL version used in the present study is relatively easy. As a matter of fact, the mean score found for our oldest participants, i.e., 30.36 (SD=3.40) was almost identical to the mean score found by Souza et al.^14^ for a group of Brazilian adults (Mean=31.5; SD=3, for the men and Mean= 28.5; SD=3, for the women). Despite this limitation, our findings confirmed the results of previous studies^15^ that age is an important predictor of variations in mental planning ability. Given the close association between age and performance in executive functions tasks,^16^ this finding is hardly surprising.

In sum, the present results strongly suggest that both biological and environmental influences are involved in the development of such systems. An important question for future research consists of investigating which specific causal factors in the environment are really important, and how they interact with biological influences to determine the growth of the neurological bases of planning ability.
