# Errratum

**DOI:** 10.1590/S1980-57642009DN20100006erratum

**Published:** 2021

In the manuscript “Planning abilities of children aged 4 years and 9 months to 8 1/2 years: Effects of age, fluid intelligence and school type on performance in the Tower of London test”, DOI: 10.1590/S1980-57642009DN20100006, published in the Dement Neuropsychol. 2008;2(1):26-30, on page 28.

**Where it reads:**

**Table 2.** Mean scores on the TOL and Raven tests as functions of age, gender, and type of school.

Age Group	Gender	Private school				Public school			
		Raven Raw score		TOL score		Raven Raw score		TOL score	
		Mean	SD	Mean	SD	Mean	SD	Mean	SD
4 years; 9 months to 5 years; 5 months; and 29 days	Girls (n = 39)	13.9	2.6	24.5	5.2	14.2	3.2	26.1	2.3
	Boys (n = 43)	17.4	2.5	28.7	2.8	2.8	2.8	2.8	3.3
	Total (n = 82)	15.4	3.1	26.4	4.7	14.1	2.4	26.4	3.0
5 years; 6 months to 6 years; 5 months; and 29 days	Girls (n = 38)	19.8	4.0	**29.4**	**29.4**	**29.4**	**29.4**	26.6	4.2
	Boys (n = 47)	19.4	3.9	29.4	3.2	15.3	2.0	26.9	5.6
	Total (n = 85)	19.5	3.9	29.4	3.2	15.8	2.4	26.8	5.0
6 years; 6 months to 7 years; 5 months; and 29 days	Girls (n = 48)	20.9	3.9	28.9	3.8	17.4	3.6	25.5	4.8
	Boys (n = 49)	22.4	4.4	28.6	3.5	17.3	2.8	27.1	4.7
	Total (n = 97)	21.7	4.2	28.8	3.6	17.3	3.2	26.4	4.8
7 years; 6 months to 8 years; 5 months; and 29 days	Girls (n = 62)	25.9	3.4	31.4	3.8	19.5	3.7	28.3	3.0
	Boys (n = 45)	25.9	3.8	31.7	2.6	20.4	4.8	28.8	2.2
	Total (n = 107)	25.9	3.6	31.5	3.2	19.8	4.0	28.5	2.8

**It should read:**

**Table 2.** Mean scores on the TOL and Raven tests as functions of age, gender, and type of school.

Age Group	Gender	Private school				Public school			
		Raven Raw score		TOL score		Raven Raw score		TOL score	
		Mean	SD	Mean	SD	Mean	SD	Mean	SD
4 years; 9 months to 5 years; 5 months; and 29 days	Girls (n = 39)	13.9	2.6	24.5	5.2	14.2	3.2	26.1	2.3
	Boys (n = 43)	17.4	2.5	28.7	2.8	2.8	2.8	2.8	3.3
	Total (n = 82)	15.4	3.1	26.4	4.7	14.1	2.4	26.4	3.0
5 years; 6 months to 6 years; 5 months; and 29 days	Girls (n = 38)	19.8	4.0	**29.4**	**3.5**	**16.5**	**2.7**	26.6	4.2
	Boys (n = 47)	19.4	3.9	29.4	3.2	15.3	2.0	26.9	5.6
	Total (n = 85)	19.5	3.9	29.4	3.2	15.8	2.4	26.8	5.0
6 years; 6 months to 7 years; 5 months; and 29 days	Girls (n = 48)	20.9	3.9	28.9	3.8	17.4	3.6	25.5	4.8
	Boys (n = 49)	22.4	4.4	28.6	3.5	17.3	2.8	27.1	4.7
	Total (n = 97)	21.7	4.2	28.8	3.6	17.3	3.2	26.4	4.8
7 years; 6 months to 8 years; 5 months; and 29 days	Girls (n = 62)	25.9	3.4	31.4	3.8	19.5	3.7	28.3	3.0
	Boys (n = 45)	25.9	3.8	31.7	2.6	20.4	4.8	28.8	2.2
	Total (n = 107)	25.9	3.6	31.5	3.2	19.8	4.0	28.5	2.8

